# Evolutionary biology and anthropology suggest biome reconstitution as a necessary approach toward dealing with immune disorders

**DOI:** 10.1093/emph/eot008

**Published:** 2013-04-19

**Authors:** William Parker, Jeff Ollerton

**Affiliations:** ^1^Department of Surgery, Duke University Medical Center, Durham, NC 27710, USA and ^2^Department of Environmental and Geographical Sciences, School of Science and Technology, University of Northampton, Newton Building, Avenue Campus, Northampton NN2 6JD, UK

**Keywords:** allergy, autoimmunity, inflammation, helminths, microbiome, mutualism, autism

## Abstract

Industrialized society currently faces a wide range of non-infectious, immune-related pandemics. These pandemics include a variety of autoimmune, inflammatory and allergic diseases that are often associated with common environmental triggers and with genetic predisposition, but that do not occur in developing societies. In this review, we briefly present the idea that these pandemics are due to a limited number of evolutionary mismatches, the most damaging being ‘biome depletion’. This particular mismatch involves the loss of species from the ecosystem of the human body, the human biome, many of which have traditionally been classified as parasites, although some may actually be commensal or even mutualistic. This view, evolved from the ‘hygiene hypothesis’, encompasses a broad ecological and evolutionary perspective that considers host-symbiont relations as plastic, changing through ecological space and evolutionary time. Fortunately, this perspective provides a blueprint, termed ‘biome reconstitution’, for disease treatment and especially for disease prevention. Biome reconstitution includes the controlled and population-wide reintroduction (i.e. domestication) of selected species that have been all but eradicated from the human biome in industrialized society and holds great promise for the elimination of pandemics of allergic, inflammatory and autoimmune diseases.

## DIAGNOSIS: PANDEMICS OF IMMUNE-RELATED DISEASE IN INDUSTRIALIZED CULTURE

Despite an ever increasing understanding of the human immune system, the field of immunology faces staggering rates of allergic, autoimmune and inflammatory diseases in industrialized societies [1, 2]. Diseases such as hay fever and food allergies, which were not described prior to 1800 and which are not found in today’s developing societies [3], are now commonplace in the USA, the UK and in other industrialized countries. With as much as 40% of the US population suffering from allergic disorders [4, 5], and another 2–8% facing autoimmune conditions [6], modern medicine has thus far failed to contain the onslaught of non-infectious, immune-related pandemics. This onslaught has contributed to a rate of chronic illness in children that approaches 50% [7] and may also be associated with a variety of cognitive disorders [8, 9] including autism [10, 11] and, as discussed below, schizophrenia. Despite this abysmal diagnosis, evolutionary biology and anthropology have provided critical information exposing a cause and subsequently a solution for these pandemics of disease. It is the purpose of this review to describe the evolutionary biology underlying this cause and to examine a view enlightened by evolutionary biology regarding a possible solution to the problem.

## EVOLUTIONARY MISMATCH AS THE UNDERLYING CAUSE OF PANDEMICS OF IMMUNE-RELATED DISEASE

Based on epidemiologic studies, cleanliness associated with modern sanitation was identified as the factor responsible for allergic diseases>20 years ago [12]. This idea, labeled the ‘hygiene hypothesis’, has been revised to the point that the term hygiene hypothesis is a complete misnomer and generally misleading [13, 14]. ‘Hygiene’, as the average industrialized citizen envisions hygiene (e.g. dust under the bed or mold in the refrigerator), is not involved [13, 15] in what is now a theoretical framework, not a hypothesis, which explains a wide range of non-infectious, immune-related diseases of industrialized society. This current model, referred to as ‘old friends’ [13] or ‘biome depletion’ [15], encompasses studies from the fields of evolutionary biology, anthropology, immunology, clinical research, basic medical research, parasitology, ecology and immunology. This model points to widely appreciated attributes of industrialized society as the causative factors underlying pandemics of immune-related disease.

The primary factor associated with allergic and autoimmune disease is apparently loss of species diversity from the ecosystem of the human body, the human biome. Species depleted or even eliminated from the human biome include a wide range of pathogens, commensals and mutualists whose reproductive cycle is greatly diminished or even eliminated by modern sanitation, water treatment and medical practices [16–18]. Importantly, the human biome, as with other biomes, not only includes species that are permanent residents of the ecosystem but also species that interact transiently with the ecosystem [19]. The absence of species from the human biome leaves the immune system in a hypersensitive state that, when combined with environmental triggers and genetic predisposition, leads to allergic and autoimmune disease. The wide range of evidence pointing incontrovertibly at this conclusion (reviewed extensively by several authors [8, 15–18, 20–22]) is summarized in Box 1. At the same time, other factors that also contribute to both allergic and autoimmune disease have been identified. For example, deprivation from sunlight as a result of indoor working environments has led to widespread vitamin D deficiency and consequently an increase in immune disease [23–26]. Other examples of factors associated with allergic and autoimmune disease are deprivation from mother’s milk [23, 25–29] and unrequited psychological stress [30–35]. Although it remains unknown whether psychological stress is increased in industrialized society, the other factors cited above are directly attributable to the culture of industrialized society. Fortunately, just as vitamin D deficiency can be compensated for by supplements, so can biome depletion be readily avoided without abandonment of modern sanitation and medicine that are necessary to avoid pandemics of various infectious diseases [15, 21].

**Box 1. Factors pointing at the importance of biome depletion in the pathogenesis of allergic and autoimmune disease**

- **Clinical observations:** Accidental helminth colonization halts the progression of multiple sclerosis [36].
- **Clinical trials:** Exposure to a porcine helminth, *T. suis*, effectively treats some patients with inflammatory bowel disease previously untreatable with modern pharmaceuticals [37].
- **Biomedical Research:** Helminths effectively avert or treat experimentally induced colitis, experimentally induced allergy and type 1 diabetes in rodent hosts [18, 91, 38–43].
- **Immunology:** (i) Helminth colonization enhances the production of regulatory elements [38, 44] that are known to reduce the propensity for allergic and autoimmune disease. (ii) Helminths are known to produce a wide range of molecules that tune down the immune system, thus decreasing the propensity for allergic and autoimmune disease [45]. (iii) Studies of both human [46] and rodent [47, 48, 49, 50] immune systems in individuals with a normal (not modified by modern technology and medicine) biome show an immune system with profoundly different regulation and a hyporesponsive posture compared with immune systems from biome-depleted individuals.
- **Evolutionary biology:** Mammalian coevolution with helminths and other species (e.g. protozoans) have resulted in ‘adjustments’ in our immune function [43] so that effective immune function is dependent on the presence of a normal biome (see text).
- **Ecology:** As with any ecosystem, profound changes in some aspects of the human biome are expected to have ramifications for many or even all other components of the biome [15].
- **Epidemiology:** The introduction of effective water treatment facilities and sewage handling systems, in combination with lingering effects of a normal biome on the immune system over decades or even generations (epigenetic effects) have created a condition in which allergic and autoimmune disease are still on the rise, but only in industrialized parts of the world.
- **Lack of alternative explanations:** Changes in breastfeeding practices, vitamin D levels and potentially psychological stress doubtless play a role in the incidence of allergic and autoimmune disease in industrialized society. However, these factors alone do not account for the widespread pandemics of allergic and autoimmune disease and, other than biome reconstitution, no other explanations are presently under consideration. Although this factor is not direct evidence for the role of biome depletion, it does underline the urgency of moving research forward at the fastest possible pace.

It is now well demonstrated if not widely appreciated that an extensive list of allergic and autoimmune diseases can be attributed to biome depletion in conjunction with other consequences of industrialized society such as vitamin D deficiency. However, the list of diseases that is associated with biome depletion is potentially much larger than previously thought (Table 1). Any disease that is associated with an immune response, generally inflammatory in nature, not associated with a genetic mutation, and lacks any apparent adaptive function, is suspect. Such diseases known to be present in industrialized but not developing societies are very highly suspect. High on the list of suspects are cognitive disorders that include autism [10, 11, 51]. Although autism is clearly a developmental disorder that may or may not be associated with ongoing aberrant immune reactivity, a number of factors point toward neuroinflammatory reactions as the factor which derailed the normal developmental process in most cases [10, 11, 52]. As described in Box 2, a wide range of cognitive disorders other than autism, including schizophrenia and migraine headaches, are associated with inflammation and may possibly be related to biome depletion. Given the potential impact of the burden of immune-related cognitive dysfunction in industrialized society, it is imperative that the possible role of biome depletion in inflammation-associated cognitive dysfunction be investigated thoroughly. Adding urgency to this mandate is the recognition that these neuroinflammatory-related diseases may be unavoidable unless steps are taken to alleviate the hypersensitive nature of the immune system in industrialized society. Further, while prevention is much more readily achieved than a cure for many allergic and autoimmune diseases [53], prevention may be the only effective option for cognitive problems associated with neurodevelopment. Once the complex milieu of biome depletion, genetics and other environmental factors have come together to induce neurodevelopmental disorders, it may be difficult if not impossible in many cases to restore health. Thus, biome reconstitution rather than treatment of disease is mandated most strongly for prevention of pandemics of inflammation-associated neurodevelopmental disorders.

**Box 2. Cognitive dysfunction as a result of biome depletion?**

- **Autism:** Becker, in 2007, was the first to point out that genetic, epidemiologic and other factors point toward autism as being associated with what is now known as biome depletion [10]. Data supporting this idea have continued to emerge [51, 52], painting a picture of autism as a disease that has biome depletion at its roots, despite vast complexity and variations in its pathological features. Although the epidemiology of autism remains a matter of contention, the strong propensity for hyperimmune reactivity in industrialized society [1, 3] and the well-established effects of inflammation on cognitive development [51] provide a rational and persuasive explanation for pandemics of autism in the absence of any other explanation. In this model, the effects of biome depletion interact with various genetic and environmental factors, leading to autism.
- **Schizophrenia:** Like autism, schizophrenia is characterized by profound cognitive dysfunction and is potentially linked to biome depletion. The association of schizophrenia with inflammation [52, 67] points toward biome depletion, as does the apparent association of schizophrenia with industrialized society [66, 75]. Schizophrenia and autism share familial links [76], and the prevalence of schizophrenia in industrialized countries is approximately 1 in 100 [75], similar to that of autism. Further, schizophrenia, like autism, is associated with infectious events during development [67] and other risk factors associated with autism [77].
- **Bipolar disorder and migraine headaches:** Their association with inflammation [63, 72] and their links with autism and schizophrenia [70, 76, 78] suggest that both bipolar disorder and migraine headaches might be yet another result of biome depletion.
- **Depression and anxiety disorders:** Both depression and anxiety disorders share two hallmarks of biome depletion-associated disease: they are associated with inflammation [14, 79–82] and affect wide swaths of the population in industrialized society.
- **Unanswered questions:** It has been argued that the increased diagnosis of at least some diseases associated with cognitive dysfunction and inflammation might reflect, at least in part, changing medical practice rather than an actual change in the incidence of disease [83, 84]. On the other hand, it is difficult to understand how a disease could be associated with inflammation, and yet not be epidemic in a society known to impose inflammation-associated diseases on the population. Unfortunately, given difficulties associated with comparing mental status over large gaps in time and culture, it seems likely that conclusive and unequivocal answers will bring an end to this debate only after biome reconstitution in humans is carried out.

**Table 1.** Some diseases associated or potentially associated with biome depletion

Disease	Confirmed in humans^a^	Supported by animal models	Industrialized^b^	Role of immunity	Role of gender	References^c^
**Confirmed or very highly probable**						
Asthma		✓	✓	✓	✓	[3]
Food allergies		✓	✓	✓	✓	[54]
Hay fever or rhinitus		✓	✓	✓	✓	[12]
Multiple sclerosis	✓	✓	✓	✓	✓	[55]
Eczema (some common types)		✓	✓	✓	✓	[56, 57]
Lupus		✓	✓	✓	✓	[6, 58]
Type 1 diabetes		✓	✓	✓	✓	[59–61]
Inflammatory bowel disease	✓	✓	✓	✓	✓	[37]
**Very probable based on role of immunity and other factors**						
Appendicitis			✓	✓	✓	[3]
Graves’ disease			✓	✓	✓	[6]
Eczema (some non-allergic types)			✓	✓	✓	[57]
Non-tropical sprue (celiac disease or gluten enteropathy)			✓	✓	✓	[62]
Migraine headaches			✓	✓	✓	[63]
Autism associated with autoantibodies			Contested	✓	✓	[11, 51]
Heart disease (in part)			✓	✓	✓	[64]
Hives (urticaria)			✓	✓	✓	[65]
Schizophrenia			✓	✓	✓	[52, 66, 67]
Dandruff			✓	✓	✓	[68]
**Suspect based on some aspects of the disease**						
Chronic fatigue syndrome			Not known	✓	✓	[69]
All autism			Contested	Contested	✓	[11]
Potential contributions to a range of neurological disorders associated with attention deficiency, bipolar behavior, anxiety, obsessive compulsiveness and depression			Additional studies needed	When known	Usually	[8, 70–72]

Contribution to inflammation associated with injury			Unknown	✓	Unknown	[73]
Psoriatic arthritis			Unknown	✓	No	[74]

^a^Confirmed in the sense that addition of helminths either reverses disease or halts the progression of disease. ^b^Associated with industrialized society more so than hunter–gatherer or traditional agrarian societies, i.e. the epidemiology is consistent with biome depletion. ^c^When applicable, the literature cited refers to papers that connect specific diseases with biome depletion. In other cases, the literature cited refers to the epidemiology of disease.

The identification of biome depletion as the cause for pandemics of immune disease carries with it a solution: biome reconstitution. Although the extent to which biome reconstitution can reverse disease remains unknown at present, biome reconstitution is hypothetically a readily available means of preventing disease associated with biome depletion. The overall goal underlying biome reconstitution is to reconstitute and maintain the biome for the prevention of disease rather than wait until treatment of disease is necessary. With this in mind, biome reconstitution is distinct from specific therapies aimed at treating disease, just as regular physical exercise is distinct from physical therapy aimed at rehabilitating a sports injury. Biome reconstitution and maintenance should be considered an important aspect of healthy living, on par with a proper diet, adequate exercise and sufficient rest. Nevertheless, biome reconstitution is certainly appropriate for biome-depleted individuals with allergic, autoimmune or other inflammatory-mediated diseases, so some overlap with therapies such as helminth therapy or microbiome transplants is expected.

## BIOME RECONSTITUTION TO COMPENSATE FOR EVOLUTIONARY MISMATCH: THE MICROBIOME

Several components of the biome are important in terms of clinical practice and reconstitution/maintenance. First, the microbiome, a subset of the biome comprised of the microorganisms of the biome, is often profoundly altered in industrialized society, rendered both abnormal and unstable by culture-specific practices. This alteration can happen initially as a result of extremely hygienic labor and delivery practices [85–87] (e.g. delivery by Caesarean section and/or cleaning of the baby with detergent after birth) or if a newborn’s mother has an altered microbiome. Subsequently, the microbiome can be profoundly altered by substituting infant formulas for breast milk [86, 88–91]. Later in life, broad spectrum antibiotics commonly used in medical practice pose a substantial risk. Further, exposure to saprophytic bacteria is greatly diminished or even lost altogether by some individuals in industrialized society. These bacteria, commonly found in soil, are still present in the environment, but cultural factors diminish or eliminate their contact with humans [16–18]. For example, prior to the widespread use of water treatment facilities, saprophytic bacteria would have been very common in virtually all drinking water and in the food supply [18]. The effects of depleting saprophytic bacteria from the human biome are unknown, although the bacteria modulate the immune system, having potent effects that can modulate emotional behavior and mood [92]. Graham Rook, with convincing, extensive and mounting evidence, has pioneered the view that that exposure to these bacteria should be an important element of biome reconstitution [17].

Constitution, reconstitution (if needed) and maintenance of the microbiome as a whole are undoubtedly vitally important for biome normalcy. Practices that alter the microbiome such as treatment with antibiotics or the use of infant formulas as a substitute for breastmilk are associated with both allergic and autoimmune disease [93–98]. Some progress is being made toward maintaining the microbiome. Advances in implementing microbiome-friendly birthing practices are being made in some parts of the world, and most medical centers are strongly encouraging breast feeding, with some success. At the same time, colonic microbiome transplants have proven extremely successful at reconstituting the colonic flora of people whose colonic microbiomes have become dramatically altered following treatment with antibiotics. In particular, recurrent *Clostridium difficile* colitis, a debilitating disease associated with alteration of the microbiome following use of broad spectrum antibiotics, has proven much more treatable with colonic microbiome transplants than with other available treatments [99–104]. Despite striking and undisputed successes with colonic microbiome transplants, use of the transplants is not yet widespread [99], and the reconstitution of other microbiome compartments, including that of the skin [105], the sinuses [106] and the vagina [107, 108], has been given no attention whatsoever in clinical practice. Further, establishment of the microbiome in newborns is seldom a priority in clinical practice. Thus, (i) current knowledge regarding reconstitution of the microbiome is not being widely utilized, (ii) exciting findings reported in the literature have seldom been translated into wide-spread practice and (iii) many potential therapeutic approaches remain unexplored. With this in mind, future efforts at biome reconstitution should consider risk factors associated with microbiome alteration (e.g. birth by Caesarean section, use of antibiotics or lack of exposure to saprophytic bacteria) and take appropriate preventative measures to avoid the consequences of microbiome alteration.

## BIOME RECONSTITUTION TO COMPENSATE FOR EVOLUTIONARY MISMATCH: EUKARYOTIC ORGANISMS

**Figure 1.**
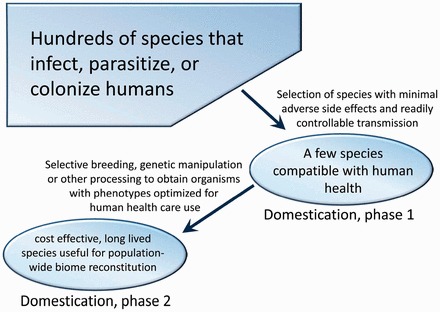
Selection and cultivation of a limited number of candidates for ‘biome reconstitution’ from a very broad array of organisms which colonize humans. A wide range of organisms, including those that cause dangerous infectious diseases, could potentially ‘stabilize’ the immune system so that it does not cause allergic and autoimmune disease. However, for biome reconstitution, only those organisms are of interest for which (a) the rate of colonization can be easily controlled, and (b) no severe adverse side effects are observed at levels that stabilize the immune system. Subsequent to this initial selection process, selective breeding, genetic manipulation or other approaches (e.g. sterilization to prevent reproduction or technological innovations to facilitate shipping and storage) may serve as a second round of the domestication process to obtain more optimal domestic species. In this manner, the proposed domestication of helminths parallels the apparent pathway by which canines were domesticated by humans [113]

Although the microbiome is sometimes destabilized or altered in industrialized cultures, other components of the biome have fared far, far worse. A wide range of eukaryotic pathogens, parasites, commensals and (potential—see below) mutualists that were once ubiquitous in humanity have been all but annihilated in industrialized populations. These organisms, which include a variety of pathogenic protozoans and helminths, cannot survive in the face of modern sanitation and water treatment facilities. Several lines of evidence indicate that it is these organisms that are most profoundly missed by industrialized immune systems (Box 1). To reconstitute this component of the biome, eukaryotic organisms that are best suited for preventing disease without causing adverse side effects must be selected or generated, starting from the wide range of species that the human immune system has coexisted with during its evolutionary history. This approach equates to domesticating a very limited number of species for the purpose of human health (Fig. 1). This idea has been described previously [15, 21] and involves selecting species, most likely helminths, which have properties that make them suitable for biome reconstitution as follows:

1. Should have negligible adverse side effects.
2. Should not reproduce under conditions of industrialized society.
3. A single exposure or at most a very limited number of exposures should have effects that last for decades, as with vaccines. This condition probably dictates that long-term colonization needs to be established. The importance of this condition for public health cannot be underestimated given that a significant percentage of the population in industrialized society is underserved by the medical community, having limited contact with medical professionals for the prevention and treatment of disease [109–111].
4. The colonization should be readily reversible, if need be.
5. It must be cost-effective. Treating the entire industrialized population will dictate that, as with vaccines, the cost of treatment for a single individual must be relatively insignificant.

Fortunately, colonization (not ‘infection’, which designates a pathogenic process) with helminths looks like a reasonable choice as a starting point for treating patients, although no one helminth necessarily fits all of the ‘ideal’ criteria described above (Box 3). The porcine whipworm (*Trichuris suis*: definitive host = *Sus scrofa*) has undergone the most testing in humans [112] and has proven useful in treating some inflammatory diseases. However, this species is clearly unsuitable for biome reconstitution, which is aimed primarily at ‘preventing’ (as opposed to treating) disease in the human population. The critical limitation inherent in the porcine whipworm is the fact that the porcine whipworm does not effectively colonize humans, and any immunomodulatory effect is temporary. Thus, exposure must be repeated on a regular basis, as often as once every 2 weeks. Other limitations of the porcine whipworm include the fact that the organisms must be isolated from pigs, which are expensive to maintain in pathogen-free conditions. With this in mind, reconstituting the biome of a majority of the human population with this species is not feasible.

**Box 3. Potential helminths for biome reconstitution**

- The ‘rat tapeworm’ (*Hymenolepis diminuta*: definitive host = *Rattus norvegicus*, with *H. sapiens* as a potential substitute; intermediate hosts = arthropods) has no adverse side effects in humans [114, 115]. The view that this helminth might help treat autoimmune disease is supported by the observation that exposure to this helminth elicits an increase in eosinophil counts [115], which is a hallmark of helminth colonization that abrogates multiple sclerosis in humans [55]. The rat tapeworm has the advantage that it can be cultivated in clean laboratory rodents and in grain beetles, components of which are already (unavoidably and harmlessly) present in the human food supply [116]. The disadvantage of the rat tapeworm is that it may require repeated exposures to have a long-term beneficial effect. Further, the rat tapeworm may not colonize immunocompetent adult humans well [115], and the lifespan of the helminth is limited to a few years. Thus, long-term treatment with a single dose of the rat tapeworm seems unlikely.
- Potentially accommodating the need for long-term colonization is the ‘bovine tapeworm’ (*Taenia saginata*; definitive host = *H. sapiens*, intermediate host = *Bos taurus*), which can readily survive in humans for >20 years. Although the bovine tapeworm is considered a commensal (non-detrimental) in humans [117], it produces egg sacks (proglottids) that are motile and thus present a potential psychological barrier to their use. Thus, it is expected that modification of the bovine tapeworm, either by genetic manipulation or by selection of naturally occurring variants, so that eggs or non-motile egg sacs rather than motile egg sacs are released from the host, will greatly increase the potential utility of the bovine tapeworm in humans.
- Another species already undergoing clinical trials [118, 119] is the ‘human hookworm’ (*Necator americanus*; host = *H. sapiens*, with incubation in soil required between hosts for completion of its life cycle). Like the rat tapeworm, this organism has a limited lifespan and thus may require repeated exposure.

Although use of the porcine whipworm in its naturally occurring form is inadequate for biome reconstitution, other approaches (Fig. 1 and Box 3) hold great promise for the future of clinical immunology. The first step is to find the most useful naturally occurring organisms in terms of effective treatment/prevention of disease, lack of adverse side effects, ability to control colonization rate and feasibility of treatment. A second phase of biome reconstitution might utilize modified organisms. For example, studies directed at understanding the longevity of parasitic worms [120], now aimed at producing drugs to eradicate the parasites, could, in addition, conceivably be used in efforts to extend the longevity of mutualistic helminths.

### EVOLUTION OF OUR CONNECTION WITH OUR COEVOLUTIONARY PARTNERS

A wide appreciation for the nature of the relationship between humans and the helminths that they host is potentially a critical factor in gaining acceptance from the medical community and indeed the public for biome reconstitution. Understanding this complex relationship requires a broader comprehension of the ecology and evolutionary biology of interspecific relationships. Interactions between wholly unrelated species are a ubiquitous feature of the Earth’s biodiversity, and all organisms interact with individuals of other species for at least part (and frequently for all) of their lifespans. The outcome of these interactions can be assessed in terms of the effect that it has on an organism’s Darwinian fitness, i.e. its ability to reproduce and the quality and quantity of its offspring.

Organismal biologists have traditionally categorized interactions between species in rather fixed terms, i.e. species *a* parasitizes species *b*; species *x* and y are mutualistic partners and so forth. This is fine as a first approximation, but in many cases, the nature of the interaction, defined by its effect on Darwinian fitness, are not fixed but are context dependent [121]. For example, almost 88% of the 350 000 species of flowering plants are biotically pollinated and use bees, butterflies, birds and other animals to disperse their pollen [122]. In most circumstances, this is a textbook example of a mutualistic relationship in which the pollinator gains a reward (usually food in the form of nectar or pollen) and the sexual reproduction of the plant is assured. But not all individual flower-visiting animals carry pollen, or are large enough to contact the sexual parts of a flower, or behave in a manner that will ensure that pollination takes place. In such circumstances, the relationship changes to a parasitic one because, while the flower visitor obtains food, the plant loses resources without being pollinated. In another example, 80% of all land plants are thought to have mycorrhizal relationships with fungi [123] in which the fungus passes water and nutrients from the soil to the plant’s roots, while the plant provides photosynthetically derived carbohydrates to the fungus. However, this mutualistic relationship can change to a parasitic one under some circumstances, with either the plant or the fungus providing no overall benefit to the partner [124].

The continuum between mutualistic and antagonistic interactions can be labile over evolutionary time scales as well as across ecological contexts. However, our understanding of how mutualism evolves into parasitism or vice versa, for example, is currently limited. Some patterns are evident from comparisons between related taxa, but elucidating the biological steps, and the selective pressures, underlying the evolution of these changes is not straightforward. One way to approach this is to model how ‘cheating’ and ‘cooperative’ genotypes fare when they interact with their host. Under different scenarios, the host may evolve mechanisms that accommodate the cooperator (for example, by providing access to a resource) or apply sanctions to the cheater (by withdrawing that resource) [125]. In the specific case of humans (or mammals more broadly) and helminths, the transition from parasitism to commensalism or mutualism may have involved a two-sided accommodation. One can envisage a chain of events in which genotypes of helminth species that have less of a negative impact on their host’s health (and therefore fitness) are tolerated in a commensal sense. Gradually this accommodation by the host becomes a reliance as the immune systems of some host genotypes evolve to ‘expect’ the presence of the helminths. Such an ‘expectation’ clearly involves the immune system genetically adapting to the presence of the symbionts, though the exact details are potentially vastly complex and certainly poorly understood.

Individuals of *Homo sapiens* are no different to any other organisms on the planet with respect to their interactions with other species. Some types of interaction are very rare, e.g. active predation by large animals. Others are widespread but with variable prevalence, such as parasitism by microorganisms (infectious disease). However, relationships with skin and gut colonizing microorganisms (which are at least partly commensalistic or mutualistic) are ubiquitous in all human populations [126–128]. With regard to the focus of this review, mutualisms between humans and other species are of particular interest. It has been proposed that, as well as gut and skin bacterial interactions, humans engage in mutualisms with a wide range of organisms, whether we are aware of it or not. This includes crop plants, food animals and domesticated pets, while traditional cultures have long engaged in cooperative relationships with wild animals such as honeyguides and dolphins [129, 130], and even urban societies benefit from interactions with local wildlife (e.g. humans and vultures in the Middle East [131]).

Mutualistic relationships between species have been described as ‘biological barter’ in that the species involved trade resources and/or services that are easily available to one species, but in short supply for the other [132]. Resources that are traded are hugely varied and include carbohydrates, inorganic nutrients, water and complex organic and inorganic chemicals. ‘Services’ are more restrictive in their range; gamete and offspring distribution via pollination and seed dispersal are well known, but other examples include physical defense of one organism by another (for example, anemonefish and their sea anemone hosts), cleaning relationships (for example, between fish or birds and mammals) and some forms of bioluminescence.

Where do human–helminth relationships sit within the notion of biological barter and the concept of biological interactions that fluctuate over ecological space and evolutionary time? Helminths are clearly obtaining nutrients and water from their physical host, as well as physical defense from the environment outside. So this relationship is based partly on resources and partly on defense, provided by the host. From the human host perspective, helminths have traditionally been viewed as always parasitic (e.g. [133]), and there is no doubt that under certain circumstances, helminths can have a negative effect on human health. However, in light of the biome depletion view, the interaction between helminths and humans needs to be recast as mutualistic, at least under certain conditions. In this view, the ‘assistance’ offered by the helminth in the development of a more effective (less prone to disease) immune system by the human would be categorized as a service.

### BIOME RECONSTITUTION: CONSIDERATIONS

As clinical work in this area proceeds to test the ideas described above regarding the effects of biome depletion and reconstitution on disease, several factors should be kept in mind. First, because the ecosystem of the human body has evolved with vast complexity, it seems unlikely that pharmaceutical interventions will ever prove successful in effectively treating biome depletion-associated disease. Biome reconstitution is intuitively the only available alternative if indeed biome depletion is at the root of the problem. Second, because the prevalence of conditions with high morbidity apparently associated with biome depletion is extremely high, exhaustive and systematic research is urgently needed. Third, because the effects of some pathologic immune reactions may be irreversible and/or may occur early in fetal development (e.g. autism), assessment of prophylactic normalization of the human biome is necessary.

Some concerns for patient safety might be raised as widespread biome reconstitution is considered. Indeed, we have pointed out several factors that might be counterindications for biome reconstitution, including immunosuppression caused either by immunodeficiency or by immunosuppressive drugs [15]. In addition, many questions regarding the implementation of biome reconstitution remain to be addressed (Box 4). For example, it remains unknown to what extent biome reconstitution will affect universal medical issues such as aging, vaccine efficiency and the pathogenesis of common infectious diseases such as the flu [15]. Thus, biome reconstitution, at least initially, should be managed by medical professionals. However, given (i) the vast experience pointing toward the safety of various components of the biome (e.g. certain helminths and the microbiome), (ii) the safeguards that will necessarily be put in place to prevent uncontrolled spread of infectious species (see earlier discussion) and (iii) the horrific consequences of biome depletion on human health, it seems foolhardy to further delay immediate efforts aimed at establishing biome reconstitution.

**Box 4. Unknown factors regarding biome reconstitution which deserve immediate and thorough investigation**

These factors have been described previously [21] and will focus on the use of helminths. The answer to these questions will be probably be affected by such variables as host age, pregnancy, disease state, gender, current state of the biome, state of the patient’s maternal biome and other factors.

- **Which helminths?** The species or combination of species and the dose (quantity and frequency of administration) of helminths that are safe and effective for treatment of disease must be determined.
- **Which diseases?** At present, it remains unknown which diseases can be cured or effectively treated with biome reconstitution, versus which can be prevented but not cured by biome reconstitution. In addition, it seems likely that some diseases that respond to biome reconstitution may not be anticipated. That is, some positive effects of biome reconstitution may come as a surprise to the medical community.
- **Prophylaxis?** Although it is anticipated that prevention of disease with biome reconstitution will prove easier than treatment, the requirements necessary to prevent disease will also need to be evaluated, especially for the purpose of preventing neurodevelopmental disorders.
- **Which patients?** The risks versus the potential benefits for reconstituting the biome of patients who have medical conditions (e.g. suppressed immune system, anemia and coagulopathy) that might make biome reconstitution more risky need to be determined.
- **Individualized medicine?** Whether biome reconstitution can be individualized may deserve attention (i.e. can biome reconstitution be tailored to the genotype of the patient?).
- **Unexpected effects?** The effect of biome reconstitution on human biology will need to be monitored carefully, since it may have an impact on a wide array of medical issues. For example, biome reconstitution might alter the efficacy of medical tools such as vaccines and immunosuppressive drugs, and might affect processes such as aging and resistance to infectious disease.

The level of difficulty that might be encountered when normalizing the biome is worth consideration. Fortunately, data from studies in laboratory rodents suggest that prevention of a wide range of allergic and autoimmune diseases may be achieved using a range of organisms [53]. Even more encouraging was the observation that colonization with a variety of helminths was sufficient to halt the progression of multiple sclerosis in humans [36]. In other words, no one particular helminth was necessary: the rules for reconstitution are apparently flexible. Consistent with this view, data from the analysis of immunity in wild rodents [47, 48] indicate that the ‘normal’ immune system, one free of the influences of modern medicine and water treatment technology, is highly variable depending on the environment. Thus, ‘normal’ immunity probably covers a very wide range, again suggesting that the rules for biome reconstitution are quite flexible. Thus, any one of a wide range of organisms or combination of such organisms might be adequate for biome reconstitution and the prevention of disease. Again, however, efforts to treat or cure disease are expected to prove more difficult than prevention [53], at least in some cases.

**Figure 2.**
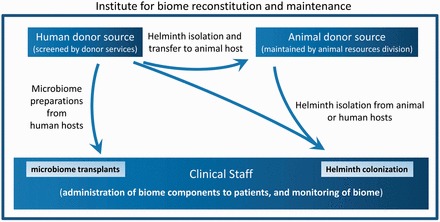
A hypothetical ‘Biome Reconstitution and Maintenance Institute’, the necessary components of which are all readily available at any major medical center today. The development of a biome reconstitution institute or center requires only reassignment of available components to new tasks. It is expected that procedures similar or identical to those already established will be used to accredit clinical laboratories, evaluate experimental treatments and provide oversight for animal use. This hypothetical diagram shows the inclusion of microbiome transplants (e.g. colonic microbiome transplants) as well as colonization with both human-derived and animal-derived helminths as a part of a center. However, in practice, microbiome transplants may be relegated to a different area, the use of helminths from more than one source may prove unnecessary, and other components of the biome (e.g. saprophytic bacteria) may also be utilized by the center for biome reconstitution

Fortunately, the actual components of a ‘Biome Reconstitution Center’ (Fig. 2) can readily be assembled at any major medical center with research capabilities. These components are in fact already present and available at any major academic medical center, although they typically do not work together for patient treatment. These necessary components include (i) the ability to screen donors, either human or non-human as needed depending on the organisms utilized for biome reconstitution, (ii) animal housing facilities as needed depending on the organisms utilized for biome reconstitution, (iii) appropriate review boards to ensure patient safety, (iv) clinical laboratories to safely purify the organisms for biome reconstitution and (v) the administrative and medical personnel to treat and monitor patients.

The field of clinical immunology is arguably the medical field which has the most to gain from an appreciation for the evolution of *H. sapiens*, and particularly the coevolution of the multiple species which comprise the human biome. Although the effects of altered diet and exercise in industrialized culture are evident and well understood from an evolutionary perspective, prevention of these effects is mired in issues involving patient education, socialization and compliance. In contrast, the field of clinical immunology can be energized and enabled in a manner that treats patients effectively with biome reconstitution to avoid allergic and autoimmune diseases, just as patients are treated effectively with vaccines to prevent infectious disease.

Some might argue that the widespread application of biome reconstitution will be difficult to achieve quickly. Current medical infrastructure is largely focused on development of patent-protected therapeutics controlled by companies with vast financial investments [53] and is aimed at treatment of individual diseases, not on prevention of wide swaths of diseases [53]. Biome reconstitution, in contrast, holds a promise for exposure of all individuals to naturally occurring organisms or selected variants of those organisms in a way that is required for human health. Such exposure must be considered a fundamental human right worthy of government support rather than an option for pharmaceutical development. This dichotomy and the fact that paradigms in science and medicine are slow to change [134] might suggest that biome reconstitution is a dream for the distant future. However, with the heavy burden of disease as a driving force, a ‘tipping point’ might be quickly reached after initial successes of pioneers in the field [21]. This view points toward a bright and near future for both biome reconstitution and clinical immunology.

## CONCLUSIONS

Evolutionary mismatches have left the typical immune system of industrialized humans prone to a wide range of allergic, autoimmune and inflammatory disease. Primary among these mismatches is the loss of helminths from the human biome. An improved understanding of the helminth/host relationship is likely important for clinicians and the public alike to accept biome reconstitution, or the reintroduction of mutualistic species into the human biome. In this manner, medical practice can accommodate the limitations of our genes as imposed by our evolutionary history, which will lead to dramatically improved public health and a revitalization of the field of clinical immunology.

**Conflict of interest**: None declared.
